# Pathways to preterm birth: Case definition and guidelines for data collection, analysis, and presentation of immunization safety data^☆^

**DOI:** 10.1016/j.vaccine.2016.03.054

**Published:** 2016-12-01

**Authors:** Margo S. Harrison, Linda O. Eckert, Clare Cutland, Michael Gravett, Diane M. Harper, Elizabeth M. McClure, Anthony P. Nunes, Suzette Lazo, Tamala Mallett Moore, Wendy Watson, Sonali Kochhar, Robert L. Goldenberg, The Brighton Collaboration Pathways to Preterm Birth Working Group

**Affiliations:** a Columbia University Medical Center, United States; b University of Washington, United States; c University of Witwatersrand, South Africa; d Global Alliance to Prevent Prematurity and Stillbirth (GAPPS), an Initiative of Seattle Childrens, United States; e University of Louisville, United States; f Research Triangle Institute, United States; g Optum Life Sciences, United States; h Philippine Society of Experimental and Clinical Pharmacology, Philippines; i Sanofi Pasteur, United States; j Pfizer, United States; k Global Healthcare Consulting, India

**Keywords:** Preterm birth, Preterm premature rupture of membranes, Preterm labor, Provider-initiated preterm birth, Insufficient cervix, Adverse event, Immunization, Guidelines, Case definition

## 1 Preamble

### 1.1 Need for developing case definitions and guidelines for data collection, analysis, and presentation for pathways to preterm birth as an adverse event following immunization

While immunizations confer protection against specific subtypes of infections, it is important to determine whether these interventions contribute to adverse maternal or neonatal outcomes in pregnancy. In particular, understanding the risk/benefit of immunizations with respect to pathways resulting in preterm birth is of particular interest. Preterm birth results from a variety of pathways ranging from idiopathic to spontaneous etiologies. Because there is a diverse spectrum of possible causes of preterm birth, it is important to determine whether some of the pathways to preterm birth can be activated by interventions, including immunizations.

The pathophysiology and pathways related to preterm birth represent diverse and complex processes. A critical principle is that spontaneous preterm births result from a spectrum of pathological processes that are initiated by specific molecular pathways, converging into a common pathway [1]. These molecular mechanisms are influenced by genetic, epigenetic, biological, behavioral, social, clinical, and environmental risk factors [1]. Different insults, such as stress, inflammation or infection, hemorrhage, uterine distention, and immune dysregulation can lead to uterine decidual and fetal membrane activation, which stimulate release of prostaglandins, cytokines, and matrix metalloproteinases that in turn lead to uterine contractions, cervical ripening, membrane rupture and subsequently, preterm birth [1]. In essence, it appears that the processes whereby term labor is initiated are implicated in preterm birth as well, with an important distinction being that term parturition results from physiologic activation of the components of the common pathway, while preterm labor arises from pathological processes that activate one or more of those components [1]. It is hypothesized that before 32 weeks gestation, the initiation of pathways to preterm birth requires a stronger stimulus than after 32 weeks, as later in the third trimester there is normal physiologic preparation of the uterus and cervix for delivery [1].

While preterm birth is being formally defined by another Brighton Collaboration working group and that definition will be incorporated into this document, the purpose of this exercise is to develop case definitions and guidelines for data collection, analysis, and presentation for the pathways to preterm birth. The etiologies of preterm birth can be grouped into four pathways that are considered the underlying events leading to preterm birth; they include: (1) premature preterm rupture of membranes (PPROM), (2) spontaneous preterm labor (PTL), (3) insufficient cervix (IC), and (4) provider-initiated preterm birth (PIPTB). Of note, the first three pathways are spontaneously occurring processes, while the fourth occurs when a decision is reached between a patient and her provider that iatrogenic initiation of labor is required for the health of the fetus, mother, or both. These four pathways are the adverse events of interest in this document, which will focus primarily on how researchers, in the context of vaccine trials, can determine that one of these four pathways has been activated, or an adverse event has occurred.

The diverse array of etiologies, coupled with obscured and/or overlapping clinical and scientific definitions has led to inconsistent definitions of pathways leading to preterm birth. Moving toward a consensus definition is critical for the purposes of monitoring adverse events in vaccine trials and to standardize terminology for improved data collection. Issues related to defining and classifying preterm birth include: relying on gestational age estimates based on a variety of approaches and validity, distinguishing between clinical versus etiologic phenotypes of preterm birth, considering whether to include or exclude multi-fetal gestations or stillborn infants, and deciding how to separate or combine different pathways to preterm birth [2]. Given that the term pathways to preterm birth has met with such difficulty in definition and classification previously, it is a crucial adverse outcome for the Brighton Collaboration to clarify for use in the context of vaccine related research.

Literature searches related to immunization in pregnancy were performed to identify existing definitions and contributing factors for premature preterm rupture of membranes, preterm labor, insufficient cervix, and provider-initiated preterm delivery; the four pathways to preterm birth. For premature preterm rupture of membranes, no data were published on the incidence of this outcome in association with immunizations in pregnancy. The same was true for insufficient cervix and provider-initiated preterm delivery; no published incidence of these outcomes related to immunization in pregnancy were available, even when similar terms were included in the search (incompetent cervix, iatrogenic preterm delivery/birth). In the case of preterm labor, while almost 100 studies were found based on the search terms preterm labor and immunization, only three of the articles mentioned preterm labor in the text of the articles, with only one reporting the incidence of preterm labor in the setting of an immunization. That paper reported that following administration of influenza A (H1N1) 2009 monovalent vaccine, 294 adverse events in pregnant women were reported to the Vaccine Adverse Event Reporting System; two women experienced preterm labor, or 1% of the immunized population in the United States [3]. There was no description of the definition used for preterm labor in this study or the long-term outcomes of those pregnancies; overall, however, it was determined that preterm labor was not likely due to the immunization itself [3].

Existing case definitions for the term, ‘pathways to preterm birth’, do not exist. Description of preterm birth as a syndrome, however, is a common finding, and attempts have been made at standardizing the pathways to preterm birth [1], [2]. One classification system published in the American Journal of Obstetrics and Gynecology proposed a method based on clinical phenotypes defined by characteristics of the mother, fetus, placenta, signs of parturition, and the pathway to delivery [2]. This methodology involves detailed information on maternal, fetal, and placental conditions as well as information regarding the initiation of parturition and the pathway to delivery [2]. Under this system a particular patient may fall into one or more of the phenotypes allowing her case to be defined by all relevant conditions, instead of forced into one strictly defined pathway [2]. For example, maternal conditions include such clinical scenarios as intra-amniotic infection, trauma, pre-eclampsia/eclampsia, and uterine rupture [2]. Fetal and placental conditions as well as symptoms of parturition and the pathway to delivery also are listed in the document [2]. While this methodology has not been validated, the authors suggest that it be piloted in a population and evaluated for utility [2]. This methodology offers a very flexible approach to the concept of classifying pathways to preterm birth, but for the purposes of this document, the goal was to develop a better-defined set of pathways to preterm birth that can be measured and documented in vaccine research.

Each of the four pathways to preterm birth as presented in this document do have existing case definitions in the literature. For example, premature preterm rupture of membranes has been defined as, “rupture of the fetal membranes before term and outside of the context of labor” [4]. Preterm labor has previously been defined as “a syndrome attributable to multiple pathologic processes leading to uterine contractions that cause cervical change before term” [6]. Insufficient cervix has been defined as, “the inability of the uterine cervix to retain a pregnancy in the absence of the signs and symptoms of clinical contractions, or labor, or both in the second trimester” [7]. For the final pathway to preterm birth, a definition published regarding what was termed ‘iatrogenic preterm birth’ (which for the purposes of this document has been called, ‘provider-initiated preterm birth’) is, “nonspontaneous delivery before term” [8]. While some of these definitions are more specific than others, they are overall too general for use as outcomes of interest in vaccine-specific and other research, and would benefit from further development as broadly applicable and clinically specific definitions. As mentioned, the term ‘pathways to preterm birth’ suffers from the lack of a formal definition and represents a missed opportunity, as data comparability across trials or surveillance systems would facilitate data interpretation and promote the scientific understanding of the event.

### 1.2 Methods for the development of the case definition and guidelines for data collection, analysis, and presentation for pathways to preterm birth as an adverse events following immunization

Following the process described on the Brighton Collaboration Website http://www.brightoncollaboration.org/internet/en/index/process.html, the Brighton Collaboration *Pathways to Preterm Birth Working Group* was formed in 2015 and included members from clinical, academic, public health, and industry backgrounds. The composition of the working and reference group as well as results of the web-based survey completed by the reference group with subsequent discussions in the working group can be viewed at: http://www.brightoncollaboration.org/internet/en/index/working_groups.html.

To guide the decision-making for the case definition and guidelines, a literature search was performed using Medline, Embase and the Cochrane Libraries, including the terms: premature preterm rupture of membranes, preterm labor, insufficient or incompetent cervix, and immunization. The search resulted in the identification of 37 references. All abstracts were screened for possible reports of initiation of the pathways to preterm birth following immunization. All articles with potentially relevant material were reviewed in more detail, in order to identify studies using case definitions or, in their absence, providing clinical descriptions of the case material. This review resulted in a detailed summary of three articles, including information on the study type, the vaccine, the diagnostic criteria or case definition put forth, the time interval since time of immunization, and any other symptoms. As such, other literature on the pathways to preterm birth and immunizations in pregnancy, in general, were made available to working group members, which included 28 papers.

### 1.3 Rationale for selected decisions about the case definition of pathways to preterm birth as an adverse event following immunization

#### 1.3.1 The term ‘Pathways to preterm birth’

The decision to focus on individual pathways to preterm birth as opposed to preterm labor alone was based on the expectation that immunizations may have pathway dependent effects, and that there is a clinical need to understand how immunization may influence the occurrence of an outcome. Addressing the classification of preterm birth or delineation of pathways to preterm birth approaches the question why a preterm birth occurred in the most comprehensive manner possible, addressing a topic that has achieved little consensus in the literature.

#### 1.3.2 The four specific pathways to preterm birth and why they were chosen for inclusion

Preterm birth is a complex syndrome that more easily defies definitional terms than conforms to them. Preterm birth is the only healthcare concept defined by an arbitrary time point rather than by a specific etiology or pathophysiology [2]. In the clinical context, and especially in the context of vaccine research, providing guidance regarding pathways to preterm birth will improve data comparability and interpretation, and as a result, improve our understanding of preterm birth and its etiologies. As such, the spontaneous preterm births were divided into the broadest three pathways that would maintain simplicity and clarity in the definition while still being comprehensive. These include (1) premature preterm rupture of membranes, (2) preterm labor, and (3) insufficient cervix. Authors have previously suggested other clinical classification methods, but generally those pathways can be combined into the aforementioned three pathways without any significant loss of specificity in determination of the correct definitional pathway. The final pathway represents non-spontaneous deliveries, and is termed (4) provider-initiated preterm birth.

#### 1.3.3 Classic features of pathways

##### 1.3.3.1 Premature preterm rupture of membranes

The term ‘fetal membranes’, or ‘membranes’, which are subject to rupture or tear either spontaneously or artificially during labor, refers to the chorion and amnion, two separate but juxtaposed layers that enclose the amniotic cavity [4]. These membranes function to contain and regulate amniotic fluid volume around the fetus, transport molecules selectively, and provide a barrier function to vaginal flora [4]. In 1–2% of pregnancies, membrane rupture preterm and outside the context of preterm labor, leads to preterm birth (about one third of all preterm births) and subsequent maternal and neonatal morbidity and mortality from infection and neonatal complications of prematurity [4]. Clinical conditions associated with PPROM include infection and/or inflammation, abruption, uterine over-distention (from multiple gestation or polyhydramnios), genetic predispositions, and tobacco use [4]. Molecular pathways implicated in PPROM include activation of matrix metalloproteinases, cytokines, apoptosis, and oxidative stress as the primary mechanisms leading to premature degradation of the fetal membranes [4], [5]. This is separate and distinct from rupture of membranes that may occur during the term labor process whereby biomechanical stress and the molecular pathways leading to membrane degradation are activated physiologically, as opposed to in the setting of PPROM, where they are activated pathologically and outside the context of labor.

##### 1.3.3.2 Preterm labor

Much like PTB and PPROM, PTL is also a syndrome associated with multiple mechanisms of disease. Analogous to PPROM, PTL is not simply activation of the mechanisms leading to labor in the preterm setting; rather, labor at term is the physiologic activation of parturition while preterm labor is the result of several possible disease processes and external stimuli leading to pathologic activation of the component pathways leading to labor and delivery [6]. Term labor is characterized by increased myometrial contractility, cervical dilation, and rupture of the chorioamniotic membranes, which are associated with a change in nuclear progesterone receptor isoforms, an increase in estrogen receptor signaling, changes in cervical extracellular matrix proteins, and decidual and membrane activation, which facilitate separation of the fetal membranes and placenta from the uterus [6]. Spontaneous preterm labor is thought to occur as a result of multiple possible disease mechanisms, including infection, vascular disorders, decidual senescence, uterine over-distention, decline in progesterone action, breakdown of maternal-fetal immune tolerance, thyroid autoimmunity, and stress, among other unknown etiologies [6]. Clinically, however, in terms of the final common pathway of labor, gestational age may be the only notable difference in settings where PTB is occurring.

##### 1.3.3.3 Insufficient cervix

In comparison to PPROM and PTL, little is known about the pathophysiology of insufficient cervix. Much of what is known about the condition is based on clinical research and associated risk factors, but less is known about the science. Factors that may increase the risk of cervical insufficiency include cervical trauma from prior surgery, such as cervical conization or loop electrosurgical excision procedures, mechanical dilation of the cervix during termination procedures, or a history of cervical laceration during delivery or prolonged labor that may damage the cervical tissue, although data confirming these associations are scant and inconsistent [9]. Currently, the practice of screening for and predicting which patients are at the greatest risk for cervical insufficiency based on history and characteristics of the incident pregnancy is the standard of care regarding prevention of preterm birth due to cervical insufficiency; unfortunately, since the presentation is usually painless cervical dilation and subsequent delivery, this pathway is often at an advanced stage when discovered and difficult, if not impossible, to treat effectively.

##### 1.3.3.4 Provider-initiated preterm birth

The global community has become focused on preterm birth and the burden it places on families, communities, countries, and healthcare systems. As such, non-spontaneous preterm births, or those that occur as a result of a healthcare provider's decision to deliver as opposed to a spontaneous process, have become a topic of particular interest because if they are avoidable, their prevention could reduce prematurity rates and complications. Review of the literature has suggested that 28–40% of all preterm deliveries are provider-initiated, and that the incidence of such deliveries may be on the rise, especially in high-income countries (HIC) [10]. Common indications for provider-initiated preterm births include maternal indications such as: hypertensive disorders, cholestasis of pregnancy, pre-existing maternal medical conditions; fetal indications, that include: anemia, alloimmunization, infection, fetomaternal hemorrhage, intrauterine growth restriction, multiple gestation with discordant fetal growth, monoamniotic twins, or twin-to-twin transfusion syndrome; and pregnancy complications, such as: placental implantation abnormalities, placental abruption, and prior uterine surgery and risk of uterine rupture [10]. Of note, the indications cited for some provider-initiated preterm births have been determined to be non-evidence-based (57% in one study), which raises concern that there is a preventable and unnecessary burden of provider-initiated preterm birth that currently exists [8].

#### 1.3.4 Standardization of diagnostics that are part of pathways definitions

Generally, diagnostics most frequently used in the levels of certainty for definitional inclusion of an event in one of the pathways to preterm birth include ultrasound and clinical evaluation. Ultrasound utilization includes determination of the amniotic fluid index as part of level 1 of certainty for PPROM, and transvaginal cervical length and dilation assessment as part of the definitions of level 1 of diagnostic certainty of preterm labor, and level 1 of diagnostic certainty for insufficient cervix. Neither amniotic fluid index nor cervical length/dilation assessment is of a particularly high level of difficulty for a routinely trained ultrasound technician in a setting where ultrasound is commonly used and regularly available. Therefore, routine training in both abdominal and transvaginal techniques should constitute standardization of ultrasound use in setting where ultrasound is highly utilized.

Clinical examination presents a challenge to standardization as it is inherently a more subjective measure and may reflect a clinician's experience and level of training. For PPROM, clinical evaluation requires the ability to collect a history, visualize leakage of fluid, and determine arborization of cervical fluid on microscopy. For PTL, clinical skills are required to quantify uterine contractions, determine rupture of membranes, and assess cervical effacement and dilation by digital examination. The diagnostic certainty of insufficient cervix also requires clinical determination of cervical effacement and dilation by digital examination and ability to collect a detailed obstetric history. The final pathway, provider-initiated preterm birth, does not require any physical clinical skills, but does require the ability to collect an obstetric history with attention to the nuance of determining whether a preterm birth was facilitated by a provider. There is no method to standardize these clinical skills; it is the hope that with high quality training and experience similar outcomes would be achieved between providers, but standardization of these clinical diagnostics are likely to exhibit inter-provider variability.

A number of commercially available products exist for diagnosing PPROM by testing vaginal discharge for amniotic fluid. These are listed as part of level 1 of diagnostic certainty for PPROM. There is no need for standardization across settings regarding which product to use, because while some products have better sensitivity and specificity than others, this is only one component of the definition, and in 90% of cases PPROM can be determined by history and gross evidence of rupture of membranes [11]. Using any one of the commercially available products should constitute standardization of diagnostic method in this circumstance.

#### 1.3.5 Settings where diagnostics may not be available

Preterm birth rates are highest in low and middle-income countries (LMIC) [12]. These are also the settings with the least resources where it may be difficult to achieve level 1 of diagnostic certainty because of difficulty with access to ultrasound or commercial amniotic fluid detection products, or because clinicians are overall less trained and/or less experienced. Overall, however, the Working Group believes that high quality data can still be collected in these settings based on levels 2 and 3 of diagnostic certainty. However, it should be noted that all levels of diagnostic certainty are considered acceptable depending on the availability of diagnostic tools in each site.

#### 1.3.6 Role of risk factors

Multiple risk factors increase a woman's risk of preterm birth outside the context of vaccine research, and should be taken into account when outcomes data are analyzed in the setting of an immunization trial in pregnancy. For example, in the United States, race and socioecomic status have been implicated as risk factors of preterm birth, with race as an independent risk factor when studies control for socioeconomic status [1]. International literature has indicated there is evidence for an increased risk of PTB for women with the following characteristics: history or family history of prior PTB, substance and tobacco use, advanced maternal age or teen pregnancy, multiple gestation and use of advanced reproductive technology, low socioeconomic status, poor prenatal care, low BMI, infections (bacterial vaginosis), poor nutrition and inappropriate weight gain during pregnancy, short interpregnancy interval, maternal hyperglycemia, persistent malarial infection, anemia (including folate deficiency), domestic violence, maternal height <145 cm, and pre-eclampsia [1]. While the pathophysiology and relationship between these risk factors and preterm birth is not always well understood, it is important to consider when analyzing data related to pathways to preterm birth.

#### 1.3.7 Formulating a case definition that reflects diagnostic certainty: weighing specificity versus sensitivity

The case definition for pathways to preterm birth has been formulated such that the Level 1 definition is highly specific for the condition. As maximum specificity normally implies a loss of sensitivity, two additional diagnostic levels have been included in the definition, offering a stepwise increase of sensitivity from Level 1 down to Level 3, while retaining an acceptable level of specificity at all levels. In this way, it is hoped that all possible cases of pathways to preterm birth can be captured.

It needs to be re-emphasized that the grading of definition levels is entirely about diagnostic certainty, not clinical severity of an event. Thus, a clinically very severe event may appropriately be classified as Level 2 or 3 rather than Level 1 if it could reasonably be of an etiology not related to a pathway leading to preterm birth. Detailed information about the severity of the event should additionally always be recorded, as specified by the data collection guidelines.

#### 1.3.8 The meaning of ‘sudden onset’ and ‘rapid progression’ in the context of pathways to preterm birth

The term “sudden onset” refers to an event that occurred unexpectedly and without warning leading to a marked change in a subject's previously stable condition. The term “rapid progression” is a conventional clinical term. An exact timeframe should not be offered since it would have to refer to a wide range of signs and symptoms without a scientific evidence base. Using an arbitrarily restrictive set point might bias future data collection unnecessarily.

#### 1.3.9 Timing post-immunization

Specific time frames for onset of symptoms following immunization are not included because pathways to preterm birth are most often activated outside the controlled setting of a clinical trial or hospital. In some settings it may be impossible to obtain a clear timeline of the event, particularly in less developed or rural settings. In order to avoid selecting against such cases, the Brighton Collaboration definition avoids setting arbitrary time frames.

#### 1.3.10 Influence of treatment on fulfillment of case definition

The Working Group decided against using ‘treatment’ or ‘treatment response’ toward fulfillment of the pathways to preterm birth case definition. A treatment response or its failure is not in itself diagnostic, and may depend on variables like clinical status, time to treatment, and other clinical parameters. Hence, the levels of diagnostic certainty definitions are designed to be broad enough to include cases presenting differently due to appropriate and early treatment initiation.

### 1.4 Guidelines for data collection, analysis and presentation

The case definitions are accompanied by guidelines, which are structured according to the steps of conducting a clinical trial, i.e. data collection, analysis and presentation. Neither case definition nor guidelines are intended to guide or establish criteria for management of ill infants, children, or adults. Both were developed to improve data comparability.

### 1.5 Periodic review

Similar to all Brighton Collaboration case definitions and guidelines, review of the definition with its guidelines is planned on a regular basis (i.e. every three to five years) or more often if needed.

## 2 Case definition of pathways to preterm birth

Pathways to preterm birth is a clinical syndrome characterized by any one or some combination of the following four pathways:

• Premature preterm rupture of membranes
• Preterm labor
• Insufficient cervix
• Provider-initiated preterm birth

For the purposes of presenting a case definition of pathways to preterm birth, each pathway will be considered individually and subject to all levels of diagnostic certainty.

### 2.1 Case definition of premature preterm rupture of membranes as a pathway to preterm birth

For all levels of diagnostic certainty:

• Patient is determined to be preterm as defined by the Brighton Collaboration definition
• On presentation, patient is determined to not be in preterm labor, having ≤4 contractions per hour documented clinically or on tocodynometer, with <2 cm cervical dilation (greater than 4 contractions per hour would qualify the patient as having preterm labor)
• Fluid can be noted to be clear, blood-tinged, meconium-tinged (fetal stool), purulent-tinged (yellowish, suggesting infection)

Level 1 Diagnostic Certainty:

1. Clinical history of rupture of membranes AND
2. Visible leakage of fluid on vaginal speculum exam AND
3. Visible arborization (ferning) on microscopy of amniotic fluid OR
4. Ultrasound with oligohydramnios (AFI <5 or MVP <2) AND
5. Documented membrane rupture by a diagnostic test (one of the below options)
  a. Positive intra-amniotic dye-injection method
  b. Positive result on amniotic fluid alpha-fetoprotein test kit
  c. Amniotic fluid pH measurement (nitrazine paper test)
  d. Amniotic fluid placental alpha macroglobulin-1 protein assay (PAMG-1) test (AmniSure test)
  e. Amniotic fluid insulin-like growth factor binding protein (IGFBP-1) test (Actim PROM test)

Level 2 Diagnostic Certainty:

1. Clinical history of rupture of membranes AND
2. Visible leakage of fluid on vaginal speculum examination AND
3. Visible arborization (ferning) on microscopy of amniotic fluid OR document membrane rupture by a diagnostic test (one of those listed above) OR
4. Ultrasound with oligohydramnios (AFI <5 or MVP <2)

Level 3 Diagnostic Certainty:

1. Clinical history of rupture of membranes AND
2. Visible leakage of presumed amniotic fluid; this may be on vaginal speculum examination (pooling in vagina), on inspection of the perineum (wet perineum due to leakage of fluid from the vagina), or fluid soaked cloth/clothes/sanitary pad

### 2.2 Case definition of preterm labor as a pathway to preterm birth

For all levels of diagnostic certainty:

• Patient is determined to be have delivered preterm as defined by the Brighton Collaboration definition

Level 1 Diagnostic Certainty:

1. On presentation, >4 documented uterine contractions per hour as determined by a tocodynometer AND
2. Documented change in length or dilation of cervix by physical examination or transvaginal ultrasound over a two hour period, with clinical criteria for documenting cervical change by exam including:
  a. Cervical dilation 2 cm or greater at the internal os by digital examination
  b. Cervical length of 1 cm or less by digital examination
  c. 50% or greater effacement by digital examination

Level 2 Diagnostic Certainty:

1. Greater than 4 uterine contractions per hour as determined by a tocodynometer or clinical assessment AND
2. Documented change in length or dilation of cervix by physical examination, with clinical criteria including:
  a. Cervical dilation 2 cm or greater at the internal os by digital examination
  b. Cervical length of 1 cm or less by digital examination
  c. 50% or greater effacement by digital examination

Level 3 Diagnostic Certainty:

1. Greater than 4 documented uterine contractions per hour determined by clinical assessment AND
2. Documented change in cervical examination (change in dilation or effacement) over a two hour period

### 2.3 Case definition of insufficient cervix as a pathway to preterm birth

For all levels of diagnostic certainty:

• Patient is determined to be ≥16 weeks and <24 weeks gestation as defined by the Brighton Collaboration definitions of gestational age
• Patient is determined to have advanced cervical dilation (>2 cm) resulting in either treatment with a cerclage (cervical stitch) or preterm delivery
• Patient is determined to not be in preterm labor, having ≤4 contractions per hour documented clinically or on tocodynometer (with anything >4 contractions per hour falling into the category of preterm labor)

Level 1 Diagnostic Certainty:

1. Internal cervical os dilation (>2 cm) with ≤4 contractions/h, as determined by transvaginal ultrasound AND digital examination

Level 2 Diagnostic Certainty:

1. Internal cervical os dilation (>2 cm) with ≤4 contractions/h, as determined by digital examination

Level 3 Diagnostic Certainty:

1. Patient reports fetal delivery without any painful contractions
2. History excludes other causes of mid-trimester delivery

### 2.4 Case definition of provider-initiated preterm delivery as a pathway to preterm birth

For all levels of diagnostic certainty:

• Patient is determined to be preterm as defined by the Brighton Collaboration definition

Level 1 Diagnostic Certainty:

1. Documentation in the healthcare record by a patient's delivering provider that there were no signs or symptoms of the spontaneous onset of preterm labor AND
2. Documentation in the healthcare record by a patient's delivering provider that the patient needed to undergo induction of labor or cesarean delivery which led to the preterm delivery

Level 2 Diagnostic Certainty:

1. From recall, delivering provider confirms that there was an absence of any signs or symptoms of the spontaneous onset of preterm labor AND
2. Delivering provider reports from recall that he or she decided that the patient needed to undergo induction of labor or cesarean delivery

Level 3 Diagnostic Certainty:

1. From recall, patient confirms that there was an absence of any signs or symptoms of the spontaneous onset of preterm labor AND
2. Patient reports from recall that the healthcare provider indicated that she needed to undergo induction of labor or cesarean delivery

## 3 Guidelines for data collection, analysis and presentation of pathways to preterm birth

It was the consensus of the Brighton Collaboration *Pathways to Preterm Birth Working Group* to recommend the following guidelines to enable meaningful and standardized collection, analysis, and presentation of information about pathways to preterm birth. However, implementation of all guidelines might not be possible in all settings. The availability of information may vary depending upon resources, geographical region, and whether the source of information is a prospective clinical trial, a post-marketing surveillance or epidemiological study, or an individual report of a pathway to preterm birth having occurred. Also, as explained in more detail in the overview paper in this volume, these guidelines have been developed by this working group for guidance only, and are not to be considered a mandatory requirement for data collection, analysis, or presentation.

### 3.1 Data collection

These guidelines represent a desirable standard for the collection of data on availability following immunization to allow for comparability of data, and are recommended as an addition to data collected for the specific study question and setting. The guidelines are not intended to guide the primary reporting of a pathway to yrs developing a data collection tool based on these data collection guidelines also need to refer to the criteria in the case definition, which are not repeated in these guidelines. The Brighton Collaboration has developed guidelines for data collection https://brightoncollaboration.org/public/resources/standards/guidelines.html; and data collection forms https://brightoncollaboration.org/public/resources/data-collection-forms.html.

Guidelines numbers below have been developed to address data elements for the collection of adverse event information as specified in general drug safety guidelines by the International Conference on Harmonization of Technical Requirements for Registration of Pharmaceuticals for Human Use, and the form for reporting of drug adverse events by the Council for International Organizations of Medical Sciences [13], [14]. These data elements include an identifiable reporter and patient, one or more prior immunizations, and a detailed description of the adverse event, in this case, of a pathway to preterm birth as having occurred following immunization. The additional guidelines have been developed as guidance for the collection of additional information to allow for a more comprehensive understanding of pathways to preterm birth that occur following immunization.

i Source of information/reporter
  For all cases and/or all study participants, as appropriate, the following information should be recorded:
  (1) Date of report.
  (2) Name and contact information of person reporting^2^ and/or diagnosing the pathways to preterm birth as specified by country-specific data protection law.
  (3) Name and contact information of the investigator responsible for the subject, as applicable.
  (4) Relation to the patient (e.g., immunizer [clinician, nurse], family member [indicate relationship], other).
ii Vaccinee/Control
  1. Demographics
    For all cases and/or all study participants, as appropriate, the following information should be recorded:
    (5) Case/study participant identifiers (e.g. first name initial followed by last name initial) or code (or in accordance with country-specific data protection laws).
    (6) Date of birth, age, and sex.
    (7) For infants: Gestational age and birth weight.
  2. Clinical and immunization history
    For all cases and/or all study participants, as appropriate, the following information should be recorded:
    (8) Past medical history, including hospitalizations, underlying diseases/disorders, pre-immunization signs and symptoms including identification of indicators for, or the absence of, a history of allergy to vaccines, vaccine components or medications; food allergy; allergic rhinitis; eczema; asthma.
    (9) Any medication history (other than treatment for the event described) prior to, during, and after immunization including prescription and non-prescription medication as well as medication or treatment with long half-life or long term effect. (e.g. immunoglobulins, blood transfusion and immunosuppressants).
    (10) Immunization history (i.e. previous immunizations and any adverse event following immunization (AEFI)), in particular occurrence of a pathway to preterm birth as having occurred after a previous immunization.
iii Details of the immunization
  For all cases and/or all study participants, as appropriate, the following information should be recorded:
  (11) Date and time of immunization(s).
  (12) Description of vaccine(s) (name of vaccine, manufacturer, lot number, dose (e.g. 0.25 mL, 0.5 mL, etc.), diluent, and number of dose if part of a series of immunizations against the same disease).
  (13) The anatomical sites (including left or right side) of all immunizations (e.g. vaccine A in proximal left lateral thigh, vaccine B in left deltoid).
  (14) Route and method of administration (e.g. intramuscular, intradermal, subcutaneous, and needle-free (including type and size), other injection devices).
  (15) Needle length and gauge.
iv The adverse event
  (16) For all cases at any level of diagnostic certainty and for reported events with insufficient evidence, the criteria fulfilled to meet the case definition should be recorded.
    Specifically document:
  (17) Clinical description of signs and symptoms of one of the pathways to preterm birth, and if there was medical confirmation of the event (i.e. patient seen by physician).
  (18) Date/time of onset,^3^ first observation^4^ and diagnosis,^5^ end of episode^6^ and final outcome.^7^
  (19) Concurrent signs, symptoms, and diseases.
  (20) Measurement/testing
    • Values and units of routinely measured parameters (e.g. temperature, blood pressure) – in particular those indicating the severity of the event;
    • Method of measurement (e.g. type of thermometer, oral or other route, duration of measurement, etc.);
    • Results of laboratory examinations, surgical and/or pathological findings and diagnoses if present.
  (21) Treatment given for pathways to preterm birth, especially any antibiotics, corticosteroids, magnesium sulfate, or tocolytics, with attention to which specific drugs were given and in what dose. Mention should be made of whether a cervical cerclage was placed in the case of insufficient cervix as a pathway to preterm birth and if so, which type.
  (22) Outcome^7^ at last observation.
  (23) Objective clinical evidence supporting classification of the event as “serious”.^8^
  (24) Exposures other than the immunization 24 h before and after immunization (e.g. food, environmental) considered potentially relevant to the reported event.
v Miscellaneous/General
  (25) The duration of surveillance for pathways to preterm birth should be predefined based on
    • Biologic characteristics of the vaccine e.g. live attenuated versus inactivated component vaccines;
    • Biologic characteristics of the vaccine-targeted disease;
    • Biologic characteristics of pathways to preterm birth including patterns identified in previous trials (e.g. early-phase trials); and
    • Biologic characteristics of the vaccines (e.g. nutrition, underlying disease like immunodepressing illness).
  (26) The duration of follow-up reported during the surveillance period should be predefined likewise. It should aim to continue to resolution of the event.
  (27) Methods of data collection should be consistent within and between study groups, if applicable.
  (28) Follow-up of cases should attempt to verify and complete the information collected as outlined in data collection guidelines 1–24.
  (29) Investigators of patients with activation of one of the pathways to preterm birth should provide guidance to reporters to optimize the quality and completeness of information provided.
  (30) Reports of activation of any of the four pathways to preterm birth should be collected throughout the study period regardless of the time elapsed between immunization and the adverse event. If this is not feasible due to the study design, the study periods during which safety data are being collected should be clearly defined.

### 3.2 Data analysis

The following guidelines represent a desirable standard for analysis of data on the occurrence of any of the pathways to preterm birth (PPROM, PTL, IC, PIPTB) to allow for comparability of data, and are recommended as an addition to data analyzed for the specific study question and setting.

(31) Reported events should be classified in one of the following five categories including the three levels of diagnostic certainty. Events that meet the case definition should be classified according to the levels of diagnostic certainty as specified in the case definition. Events that do not meet the case definition should be classified in the additional categories for analysis.

Event classification in 5 categories^9^

Event meets case definition
  (1) Level 1: Criteria as specified in the Pathways to Preterm Birth case definition
  (2) Level 2: Criteria as specified in the Pathways to Preterm Birth case definition
  (3) Level 3: Criteria as specified in the Pathways to Preterm Birth case definition
Event does not meet case definition*Additional categories for analysis*
  (4) Reported Pathways to Preterm Birth with insufficient evidence to meet the case definition^10^
  (5) Not a case of Pathways to Preterm Birth

(32) The interval between immunization and reported activation of a pathway to preterm birth could be defined as the date/time of immunization to the date/time of onset^3^ of the first symptoms and/or signs consistent with the definition. If few cases are reported, the concrete time course could be analyzed for each; for a large number of cases, data can be analyzed in the following increments:

Subjects with activation of a pathway to preterm birth by interval to presentation.

**Table:** 

Interval	Number
<24 h	
1–7 days	
8–30 days	
31 days +	
Total

(33) The duration of a possible Pathways to Preterm Birth could be analyzed as the interval between the date/time of onset^2^ of the first symptoms and/or signs consistent with the definition and the end of episode^6^ and/or final outcome^7^. Whatever start and ending are used, they should be used consistently within and across study groups.
(34) If more than one measurement of a particular criterion is taken and recorded, the value corresponding to the greatest magnitude of the adverse experience could be used as the basis for analysis. Analysis may also include other characteristics like qualitative patterns of criteria defining the event.
(35) The distribution of data (as numerator and denominator data) could be analyzed in predefined increments (e.g. measured values, times), where applicable. Increments specified above should be used. When only a small number of cases is presented, the respective values or time course can be presented individually.
(36) Data on Pathways to Preterm Birth obtained from subjects receiving a vaccine should be compared with those obtained from an appropriately selected and documented control group(s) to assess background rates of hypersensitivity in non-exposed populations, and should be analyzed by study arm and dose where possible, e.g. in prospective clinical trials.

### 3.3 Data presentation

These guidelines represent a desirable standard for the presentation and publication of data on Pathways to Preterm Birth following immunization to allow for comparability of data, and are recommended as an addition to data presented for the specific study question and setting. Additionally, it is recommended to refer to existing general guidelines for the presentation and publication of randomized controlled trials, systematic reviews, and meta-analyses of observational studies in epidemiology (e.g. statements of Consolidated Standards of Reporting Trials (CONSORT), of Improving the quality of reports of meta-analyses of randomized controlled trials (QUORUM), and of Meta-analysis Of Observational Studies in Epidemiology (MOOSE), respectively) [15], [16], [17].

(37) All reported events of Pathways to Preterm Birth should be presented according to the categories listed in guideline 31.
(38) Data on possible Pathways to Preterm Birth events should be presented in accordance with data collection guidelines 1–24 and data analysis guidelines 31–36.
(39) Terms to describe Pathways to Preterm Birth such as “low-grade”, “mild”, “moderate”, “high”, “severe” or “significant” are highly subjective, prone to wide interpretation, and should be avoided, unless clearly defined.
(40) Data should be presented with numerator and denominator (*n*/*N*) (and not only in percentages), if available.
  Although immunization safety surveillance systems denominator data are usually not readily available, attempts should be made to identify approximate denominators. The source of the denominator data should be reported and calculations of estimates be described (e.g. manufacturer data like total doses distributed, reporting through Ministry of Health, coverage/population based data, etc.).
(41) The incidence of cases in the study population should be presented and clearly identified as such in the text.
(42) If the distribution of data is skewed, median and range are usually the more appropriate statistical descriptors than a mean. However, the mean and standard deviation should also be provided.
(43) Any publication of data on Pathways to Preterm Birth should include a detailed description of the methods used for data collection and analysis as possible. It is essential to specify:
  • The study design;
  • The method, frequency and duration of monitoring for activation of Pathways to Preterm Birth;
  • The trial profile, indicating participant flow during a study including drop-outs and withdrawals to indicate the size and nature of the respective groups under investigation;
  • The type of surveillance (e.g. passive or active surveillance);
  • The characteristics of the surveillance system (e.g. population served, mode of report solicitation);
  • The search strategy in surveillance databases;
  • Comparison group(s), if used for analysis;
  • The instrument of data collection (e.g. standardized questionnaire, diary card, report form);
  • Whether the day of immunization was considered “day one” or “day zero” in the analysis;
  • Whether the date of onset^3^ and/or the date of first observation^4^ and/or the date of diagnosis^5^ was used for analysis; and
  • Use of this case definition for Pathways to Preterm Birth, in the abstract or methods section of a publication^11^,^12^.

## Footnotes

2 If the reporting center is different from the vaccinating center, appropriate and timely communication of the adverse event should occur.

3 The date and/or time of onset is defined as the time post immunization, when the first sign or symptom indicative for Pathways to Preterm Birth occurred. This may only be possible to determine in retrospect.

4 The date and/or time of first observation of the first sign or symptom indicative for pathways to preterm birth can be used if date/time of onset is not known.

5 The date of diagnosis of an episode is the day post immunization when the event met the case definition at any level.

6 The end of an episode is defined as the time the event no longer meets the case definition at the lowest level of the definition.

7 E.g. recovery to pre-immunization health status, spontaneous resolution, therapeutic intervention, persistence of the event, sequelae, death.

8 An AEFI is defined as serious by international standards if it meets one or more of the following criteria: (1) it results in death, (2) is life-threatening, (3) it requires inpatient hospitalization or results in prolongation of existing hospitalization, (4) results in persistent or significant disability/incapacity, (5) is a congenital anomaly/birth defect, (6) is a medically important event or reaction.

9 To determine the appropriate category, the user should first establish, whether a reported event meets the criteria for the lowest applicable level of diagnostic certainty, e.g. Level three. If the lowest applicable level of diagnostic certainty of the definition is met, and there is evidence that the criteria of the next higher level of diagnostic certainty are met, the event should be classified in the next category. This approach should be continued until the highest level of diagnostic certainty for a given event could be determined. If the lowest level of the case definition is not met, it should be ruled out that any of the higher levels of diagnostic certainty are met and the event should be classified in additional categories four or five.

10 If the evidence available for an event is insufficient because information is missing, such an event should be categorized as “Reported Cases of Activation of a Pathway to Preterm Birth with insufficient evidence to meet the case definition”.

11 An event does not meet the case definition if investigation reveals a negative finding of a necessary criterion (necessary condition) for diagnosis. Such an event should be rejected and classified as “Not a case of Activation of a Pathway to Preterm Birth”.

12 Use of this document should preferably be referenced by referring to the respective link on the Brighton Collaboration website (http://www.brightoncollaboration.org).
